# Triglycerides to high-density lipoprotein cholesterol ratio as a surrogate for nonalcoholic fatty liver disease: a cross-sectional study

**DOI:** 10.1186/s12944-019-0986-7

**Published:** 2019-02-02

**Authors:** Nengguang Fan, Liang Peng, Zhenhua Xia, Lijuan Zhang, Zhiyi Song, Yufan Wang, Yongde Peng

**Affiliations:** 1 Department of Endocrinology and Metabolism, Shanghai General Hospital, School of Medicine, Shanghai Jiao Tong University, 100 Haining Road, Shanghai, 200080 China; 2 Department of Endocrinology, Shanghai Songjiang Center Hospital, Shanghai, 201600 China; 3 Department of Laboratory Medicine, Shanghai Songjiang Center Hospital, Shanghai, 201600 China

**Keywords:** TG to HDL-C ratio, Nonalcoholic fatty liver disease, Insulin resistance, Epidemiology

## Abstract

**Background:**

Triglycerides (TG) to high-density lipoprotein cholesterol (HDL-C) ratio (TG/HDL-C) has been recommended as a surrogate marker for insulin resistance. In the present study, we aimed to investigate the relationship between TG/HDL-C and NAFLD in an apparently healthy population.

**Methods:**

A total of 18,061 subjects who participated in a health checkup program were included. NAFLD was diagnosed by ultrasonography.

**Results:**

The prevalence rate of NAFLD was 24.8% in the whole population, and progressively increased across the quartiles of TG/HDL-C (4.9, 14.1, 26.8 and 53.5%, respectively, *P* <  0.001). After adjustment for confounding factors, TG/HDL-C was independently associated with the risk of NAFLD. Compared with the first quartile of TG/HDL-C (Q1), the odds ratios (95% confidence intervals) for NAFLD in the increasing quartiles (Q2-Q4) were 2.1(1.8–2.6), 3.6 (3.0–4.3) and 9.2(7.6–11.1), respectively. In addition, the area under receiver operator characteristic curve (95% confidence interval) of TG/HDL-C for NAFLD was 0.85 (0.84–0.86) in women and 0.79 (0.78–0.80) in men, significantly higher than that of TG, TC, LDL-C, HDL-C, ALT and AST (*P* <  0.05). The optimal cutoff point of TG/HDL-C for detection of NAFLD was 0.9 in women (sensitivity = 78.8%, specificity = 77.3%) and 1.4 in men (sensitivity = 70.7%, specificity = 73.5%).

**Conclusions:**

TG/HDL-C is independently associated with NAFLD in apparently healthy individuals and may be used as a surrogate for NAFLD.

## Background

Nonalcoholic fatty liver disease (NAFLD), defined as the presence of hepatic steatosis in the absence of alcohol use and other causes of liver disease, has become one of the most prevalent liver diseases worldwide [1]. It represents a spectrum of conditions from simple steatosis to nonalcoholic steatohepatitis (NASH) and cirrhosis. A growing body of evidence has linked NAFLD to a range of metabolic disorders, including obesity, dyslipidemia and diabetes. Now it has been well recognized that insulin resistance plays a central role in the development of NAFLD [2].

Increased triglycerides (TG) and decreased high-density lipoprotein cholesterol (HDL-C) concentrations always appear in metabolic syndrome. In past few years, the ratio of TG/HDL-C has shown to be closely related to insulin resistance [3–5]. Compared with other lipid parameters, TG/HDL-C was the strongest correlate of the homeostasis model assessment of insulin resistance in a multiethnic primary prevention cohort study [6]. McLaughlin et al. reported that a TG/HDL-C ratio of 1.8 or greater could predict insulin resistance in Caucasians [7]. While in African Americans, a TG/HDL-C ratio of 1.2 or greater was shown to predict insulin resistance [8]. Thus, TG/HDL-C has been recommended as a surrogate for insulin resistance. Moreover, TG/HDL-C has also shown to be a predictor of type 2 diabetes, hypertension and cardiovascular diseases [9–12].

Recently, there is limited evidence available suggesting an association of TG/HDL-C with NAFLD. In a cross-sectional study including children and adolescents, higher TG/HDL-C was associated with an increased odds ratio for NAFLD [13]. More recently, a population-based historical cohort study in Japan showed that TG/HDL-C predicted the incident fatty liver and NAFLD [14]. However, further researches are warranted to confirm the relationship between TG/HDL-C and NAFLD.

In the present study, we performed a cross-sectional study to determine whether TG/HDL-C ratio is associated with NAFLD in anapparently healthy population. Furthermore, the ability of TG/HDL-C and its optimal cutoff point for detecting NAFLD was also investigated.

## Methods

### Subjects

The study population was recruited from adults who underwent health checkups at the Shanghai First People’s Hospital between May 2013 and June 2014. Subjects with an alcohol intake > 140 g/week for men and 70 g/week for women, a history of viral hepatitis, auto-immune hepatitis or other forms of chronic liver disease were excluded from the study. Finally, a total of 18,061 subjects were included in the final analysis.

### Anthropometric and biochemical measurements

All subjects were assessed after overnight fasting for at least 10 h. Body weight, height, systolic and diastolic blood pressure (SBP, DBP) were measured by an experienced physician. Body mass index (BMI) was calculated as body weight in kilograms divided by body height squared in meters.

Blood samples were collected from the cubital vein by one experienced nurse. Fasting serum triglycerides (TG), total cholesterol (TC), low-density lipoprotein cholesterol (LDL-C), high-density lipoprotein cholesterol (HDL-C), alanine aminotransferase (ALT), aspartate aminotransferase (AST), serum uric acid (SUA) and serum creatinine (Scr) were measured using an autoanalyzer (Beckman, Palo Alto, CA). Blood glucose was measured with glucose oxidase method.

### Diagnosis of NAFLD

The diagnosis of NAFLD was based on the results of abdominal ultrasonography using a high-resolution B-mode tomographic ultrasound system with a 3.5-MHz probe (Toshiba, Tokyo, Japan). According to Diagnostic Criteria of Nonalcoholic Fatty Liver Disease by the Chinese Society of Hepatology in 2010 [15], hepatic steatosis was defined by the presence of at least 2 of 3 of the following abnormal findings: diffuse hyperechogenicity of the liver relative to the kidney; attenuation of the ultrasound beam; poor visualization of intrahepatic architectural details. Alcohol consumption, viral, or autoimmune liver disease was excluded before NAFLD diagnosis.

### Statistical analysis

All statistical analyses were performed using SPSS 13.0 (Chicago, IL). Continuous variables were presented as means ± SD or median (interquartile range), and categorical variables were displayed as percentages (%). Non-normally distributed data were logarithmically transformed before analysis. The significance of differences between groups was evaluated by Student’s t test or one-way analysis of variance (ANOVA) for continuous variables and *x*^2^ test for categorical variables. Logistic regression was used to evaluate the association between TG/HDL-C and NAFLD. Receiver operator characteristic (ROC) curve analyses were performed to assess the abilities of TG/HDL-C or other biochemical parameters to detect NAFLD. *P* <  0.05 was considered statistically significant.

## Results

### Clinical characteristics of the study population

Among the 18,061 enrolled individuals, the overall prevalence rate of NAFLD was 24.8%. In comparison with the control group, subjects with NAFLD were older, more likely to be male, and had higher levels of BMI, SBP, DBP, TG, TC, LDL-C, FPG, TG/HDL-C, SUA, ALT and AST (*P* < 0.001). In contrast, HDL-C was significantly reduced in the NAFLD group (Table 1).

**Table 1:** Clinical and biochemical characteristics of the study subjects with or without NAFLD

Variables	Non-NAFLD	NAFLD	*P*
N	13,581	4480	
Sex (M/F)	5191/8390	3256/1244	< 0.001
AGE (years)	39.5 ± 10.5	45.8 ± 9.9	< 0.001
BMI (kg/m^2^)	22.1 ± 2.6	26.4 ± 2.7	< 0.001
SBP (mmHg)	114.6 ± 14.8	127.4 ± 15.7	< 0.001
DBP (mmHg)	75.4 ± 9.9	83.9 ± 10.0	< 0.001
FPG (mM)	5.2 ± 0.8	5.8 ± 1.4	< 0.001
TG (mM)	1.0 (0.7–1.5)	2.1 (1.5–3.1)	< 0.001
TC (mM)	4.7 ± 0.9	5.2 ± 1.0	< 0.001
LDL-C (mM)	2.7 ± 0.7	3.1 ± 0.8	< 0.001
HDL-C (mM)	1.5 ± 0.4	1.2 ± 0.3	< 0.001
TG/HDL-C	0.7 (0.4–1.1)	1.8 (1.2–3.0)	< 0.001
SUA (μM)	302.8 ± 79.6	386.2 ± 86.1	< 0.001
Scr (μM)	70.1 ± 15.3	78.1 ± 15.2	< 0.001
ALT (IU/L)	10 (7–13)	17 (12–25)	< 0.001
AST (IU/L)	20 (17–24)	24 (20–30)	< 0.001

Continuous variables were presented as means ± SD or median (interquartile range)

**Fig. 1:**
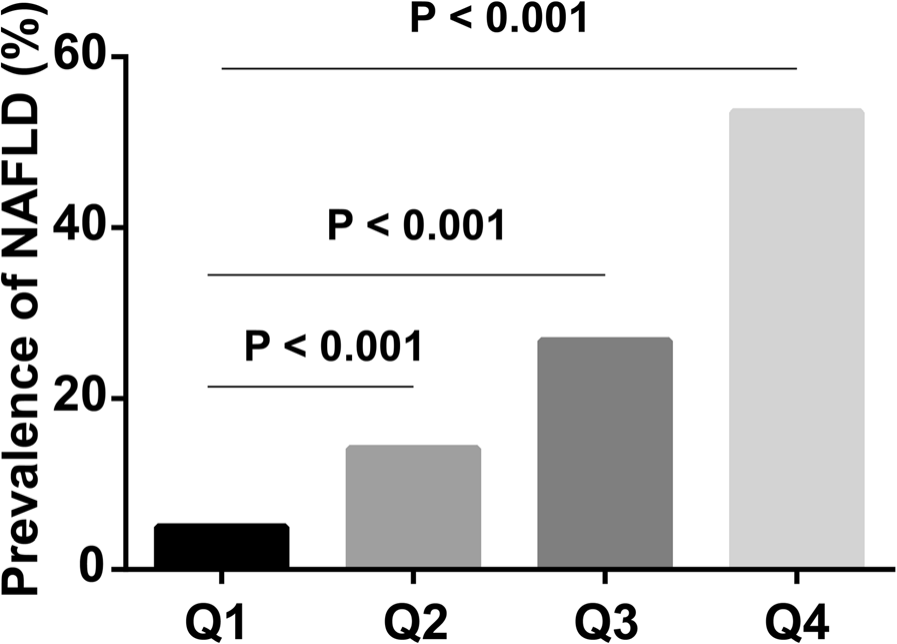
Prevalence of NAFLD across the quartiles of TG/HDL-C. The prevalence of NAFLD in four groups according sex-specific quartiles of TG/HDL-C (Q1-Q4)

Clinical and biochemical characteristics of the participants according to the quartiles of sex-specific TG/HDL-C were summarized in Table 2. With the increase of TG/HDL-C from the first to the fourth quartile (Q1-Q4), parameters including BMI, DBP, SBP, TG, TC, LDL-C, FPG, SUA, Scr, ALT and AST were all significantly elevated (all *P* for trend < 0.001). Moreover, the prevalence rates of NAFLD were also progressively increased across the quartiles of TG/HDL-C (4.9, 14.1, 26.8 and 53.5%, respectively, *P* for trend < 0.001, Fig. 1).

**Table 2:** Clinical and biochemical characteristics of the study subjects according to quartiles of TG/HDL

Characteristics	Q 1	Q 2	Q 3	Q 4	*P* for trend
n	4522	4533	4466	4535	
Age (years)	37.5 ± 10.3	39.4 ± 10.4	41.8 ± 10.4	45.5 ± 10.0	< 0.001
Male/Female	2123/2399	2107/2426	2098/2368	2115/2420	0.957
BMI (kg/m^2^)	21.5 ± 2.6	22.5 ± 2.9	23.5 ± 3.2	24.9 ± 3.1	< 0.001
SBP (mmHg)	112.6 ± 13.9	115.3 ± 15.0	118.4 ± 15.9	124.7 ± 16.6	< 0.001
DBP (mmHg)	74.4 ± 9.7	76.0 ± 10.3	78.1 ± 10.5	81.7 ± 10.3	< 0.001
FPG (mM)	5.1 ± 0.6	5.2 ± 0.9	5.4 ± 1.0	5.6 ± 1.3	< 0.001
TG (mM)	0.7 (0.6–0.9)	1.0 (0.8–1.3)	1.4 (1.1–1.9)	2.5 (1.8–3.5)	< 0.001
TC (mM)	4.5 (4.0–5.0)	4.6 (4.1–5.2)	4.8 (4.2–5.4)	5.1 (4.5–5.8)	< 0.001
LDL-C (mM)	2.5 ± 0.6	2.7 ± 0.7	2.9 ± 0.7	3.0 ± 0.8	< 0.001
HDL-C (mM)	1.8 ± 0.4	1.5 ± 0.3	1.3 ± 0.3	1.1 ± 0.2	< 0.001
ALT (IU/L)	9 (7–13)	10 (7–14)	11 (8–17)	13 (10–21)	< 0.001
AST (IU/L)	20 (18–24)	20 (17–24)	21 (18–25)	22 (19–28)	< 0.001
Scr (μM)	69.9 ± 14.9	72.5 ± 15.4	74.6 ± 15.6	78.7 ± 20.2	< 0.001
SUA (μM)	296.0 ± 77.4	311.8 ± 83.7	328.3 ± 89.1	357.7 ± 92.5	< 0.001

Continuous variables were presented as means ± SD or median (interquartile range)

### Association between TG/HDL-C and NAFLD

Logistic regression analysis was performed to determine the independent association between TG/HDL-C and risk of NAFLD. As shown in Table 3, elevating quartiles of TG/HDL-C were associated with increased risk of NAFLD in univariate analysis (model 1). After adjustment for age and sex (model 2), and further for BMI, SBP and DBP (model 3), the odds ratios (ORs) for NAFLD remained progressively increased across quartiles of TG/HDL-C. Finally, TG/HDL-C was still independently associated with the risk of NAFLD after further controlling for FPG, TG, TC, HDL-C, LDL-C and Scr (model 4). Compared with the first quartile (Q1), the ORs (95%CI) for NAFLD in the increasing quartiles of TG/HDL-C (Q2-Q4) were 2.1(1.8–2.6), 3.6 (3.0–4.3) and 9.2(7.6–11.1), respectively (Table 3).

**Table 3:** The risk of NAFLD according to quartiles of TG/HDL

	Q 1	Q2	Q 3	Q4	*P* for trend
Model 1	1	3.2	7.1	22.4	
		(2.7–3.7)	(6.1–8.3)	(19.3–25.9)	
*P* Value		< 0.001	< 0.001	< 0.001	< 0.001
Model 2	1	3.3	7.8	28.8	
		(2.9–3.9)	(6.7–9.2)	(24.6–33.7)	
*P* Value		< 0.001	< 0.001		< 0.001
Model 3	1	2.3	4.2	12.0	
		(1.9–2.8)	(3.5–5.0)	(10.0–14.5)	
*P* Value		< 0.001	< 0.001		< 0.001
Model 4	1	2.1	3.6	9.2	
		(1.8–2.6)	(3.0–4.3)	(7.6–11.1)	
*P* Value		< 0.001	< 0.001	< 0.001	< 0.001

Data are odds ratios (95% confidence interval)

Model 1 is unadjusted

Model 2 is adjusted for age, sex

Model 3 is further adjusted for BMI, SBP, DBP

Model 4 is further adjusted for FPG, TG, TC, LDL-C, HDL-C, UA and Scr

### The ability of TG/HDL-C to detect NAFLD

**Fig. 2:**
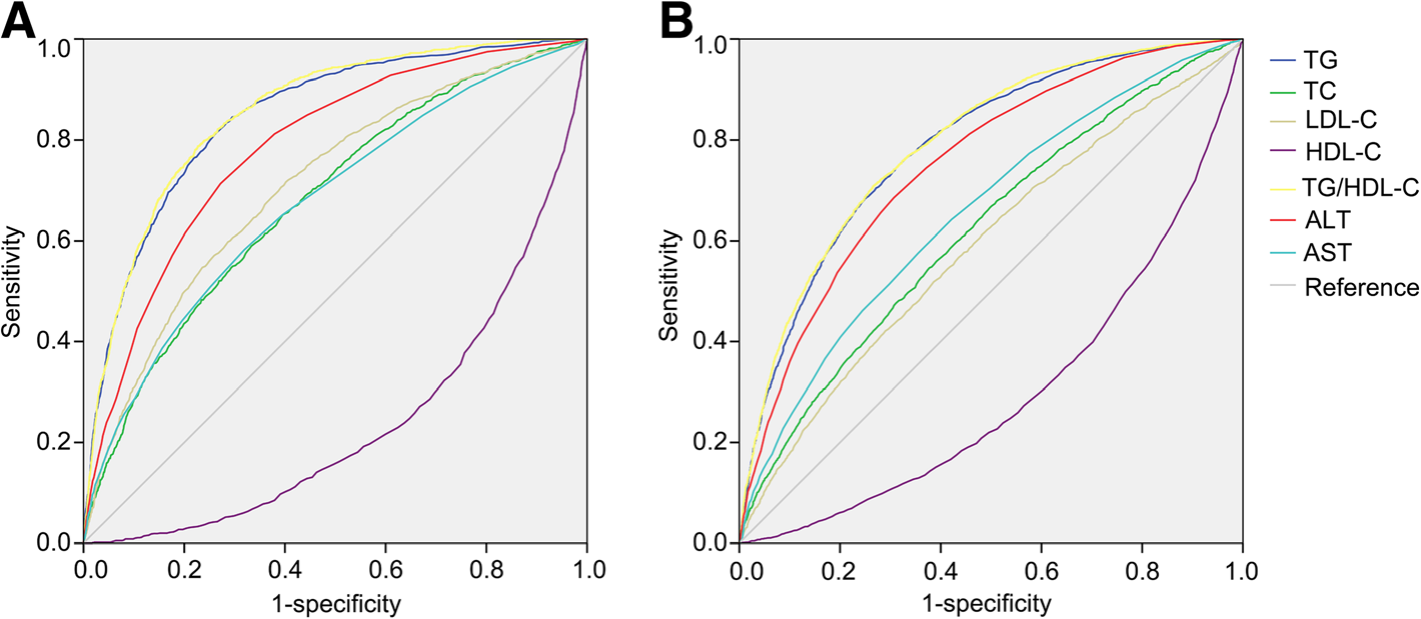
ROC curves of TG/HDL-C and other lipid components or markers of liver injury. ROC curves of TG/HDL-C, TG, TC, LDL-C, HDL-C, ALT and AST were presented in women and men. **a** ROC curves in women. **b** ROC curves in men

To investigate the ability of TG/HDL-C to detect NAFLD, the ROC curve of TG/HDL-C was analyzed and compared with that of other lipid components and markers of liver injury (ALT and AST). As shown in Table 4 and Fig. 2, the AUC of TG/HDL-C was 0.85 (0.84–0.86) in women and 0.79 (0.78–0.80) in men, significantly higher than that of TG, TC, LDL-C, HDL-C, ALT and AST (all *P* <  0.05). The optimal cutoff point of TG/HDL-C for detecting NAFLD was 0.9 in women (sensitivity = 78.8%, specificity = 77.3%) and 1.4 in men (sensitivity = 70.7%, specificity = 73.5%).

**Table 4:** Comparison of areas under the ROC curves (95% CI) of potential markers for NAFLD in subjects categorized by sex

AUC(95% CI)						
Characteristics	Total	*P* value	Women	*P* value	Men	*P* value
TG	0.84 (0.83–0.85)	< 0.001	0.84 (0.83–0.85)	< 0.001	0.78 (0.77–0.79)	< 0.001
TC	0.65 (0.64–0.65)	< 0.001	0.68 (0.67–0.69)	< 0.001	0.62 (0.61–0.63)	< 0.001
LDL-C	0.65 (0.64–0.65)	< 0.001	0.71 (0.70–0.72)	< 0.001	0.59 (0.58–0.60)	< 0.001
HDL-C	0.77 (0.76–0.78)	< 0.001	0.76 (0.75–0.77)	< 0.001	0.70 (0.69–0.71)	< 0.001
TG/HDL-C	0.85 (0.84–0.86)	< 0.001	0.85 (0.84–0.86)	< 0.001	0.79 (0.78–0.80)	< 0.001
ALT	0.80 (0.80–0.81)	< 0.001	0.78 (0.77–0.79)	< 0.001	0.75 (0.74–0.76)	< 0.001
AST	0.70 (0.69–0.70)	< 0.001	0.68 (0.67–0.69)	< 0.001	0.66 (0.65–0.67)	< 0.001

## Discussion

TG/HDL-C has shown to be a surrogate for insulin resistance, which plays a central role in the pathogenesis of NAFLD. However, the relationship between TG/HDL-C and NAFLD has not been well elucidated. In the present study, we found that TG/HDL-C was independently associated with the risk of NAFLD in an apparently healthy population. Moreover, TG/HDL-C was a better predictor of NAFLD as compared with other lipid parameters and markers of liver injury.

In the past few years, a growing body of evidence has shown an association between TG/HDL-C and more unfavorable metabolic profiles, including dyslipidemia, obesity and diabetes. Consistent to these previous results, our present study also showed significantly higher BMI, blood pressure, TG, TC, LDL-C, FPG andSUA in subjects with higher TG/HDL-C as compared to those with lower TG/HDL-C.

The association between TG/HDL-C and NAFLD was recently investigated. A cohort study showed that higher TG/HDL-C was strongly associated with increased risk of incident fatty liver and NAFLD [14]. Similarly, in a cross-sectional study involving a large sample of children and adolescents, TG/HDL-C was independently related to NAFLD [13]. Consistently, our present study also revealed a close relationship between TG/HDL-C and NAFLD. The prevalence and ORs of NAFLD increased progressively across the quartiles of TG/HDL-C. However, the ability of TG/HDL-C to predict NAFLD was not investigated in the previous studies [13, 14]. Though one study showed that the AUC of TG/HDL-C for incident fatty liver was greater than that of other lipid parameters and the optimal cut-off points of TG/HDL-C for incident fatty liver were 0.88 in men and 0.64 in women, the authors did not further study the ability of TG/HDL-C to detect NAFLD [14]. In the present study, we found that the AUC of TG/HDL-C for detecting NAFLD is 0.79 in men and 0.85 in women, significantly higher than that of other lipid parameters and markers of liver injury. Thus, TG/HDL-C may be a potential surrogate for NAFLD.

Though the mechanism underlying the link between TG/HDL-C and NAFLD has not been fully elucidated, insulin resistance is a potential mediator. TG/HDL-C was found to be closely associated with insulin resistance in different populations including Caucasians, Korean and Chinese, and has been recommended as a clinical indicator of insulin resistance [4, 5, 7, 16]. In experimental studies, insulin resistance was shown to promote the secretion of larger and TG over-enriched VLDL particles [17, 18], but decrease the concentration of HDL-C. Thus, insulin resistance contributes to the increase of TG/HDL-C. On the other hand, insulin resistance promotes NAFLD by inducing lipolysis of adipose tissue TG and de novo synthesis of TG in the liver [19, 20]. Thus, insulin resistance may be responsible for the association between TG/HDL-C and NAFLD. Due to the lack data of serum insulin level, the association between TG/HDL-C and insulin resistance was not investigated in the present study. Additionally, adiponectin may be another linker between TG/HDL-C and NAFLD. Previous studies have shown that adiponectin increases serum HDL-C and conversely lowers serum TG [21], thus reduced adiponectin may lead to increased TG/HDL-C. With regard to NAFLD, low serum adiponectin has shown to be a predictor of its progression [22]. Mechanistically, reduced adiponectin signaling contributes to NAFLD through inactivation of adenosine monophosphate activated protein kinase (AMPK) and decreased mitochondrial biogenesis and β-oxidation [23]. It warrants further investigation whether adiponectin is responsible for the correlation between TG/HDL-C and NAFLD in future studies. There are several limitations that require consideration. First, our study was cross-sectional, which did not allow to make a cause-effect inference. Second, the best method for an accurate diagnosis of NAFLD is liver biopsies. Ultrasonic examination, which was applied in the present study for diagnosis of NAFLD, is not sensitive enough to detect mild liver steatosis. However, this noninvasive method is still widely used in clinical practice and epidemiological studies and is accepted for its sensitivity and specificity in detecting hepatic steatosis. Thirdly, the influence of diet was not assessed in our study. Previous studies have shown that nutraceuticals can affect both serum lipid and NAFLD [24, 25], so diet may influence the association between TG/HDL-C and NAFLD.

## Conclusion

TG/HDL-C is independently associated with NAFLD and may be used as a surrogate for NAFLD.

## Abbreviations

ALT: alanine aminotransferase
AST: aspartate aminotransferase
AUC: area under the ROC curve
BMI: body mass index
DBP: diastolic blood pressure
HDL-C: high-density lipoprotein cholesterol
NAFLD: nonalcoholic fatty liver disease
OR: odds ratio
ROC: receiver operator characteristic
SBP: systolic blood pressure
Scr: serum creatinine
SUA: serum uric acid
TC: total cholesterol
TG: triglycerides
TG/HDL-C: TG to HDL ratio
